# Clinical and histopathologic findings in dogs with the ultrasonographic appearance of gastric muscularis unorganized hyperechoic striations

**DOI:** 10.1186/s13028-018-0365-9

**Published:** 2018-02-09

**Authors:** Hock Gan Heng, Chee Kin Lim, Sarah Steinbach, Meaghan Maureen Broman, Margaret Allan Miller

**Affiliations:** 1 Department of Veterinary Clinical Sciences, College of Veterinary Medicine, Purdue University, West Lafayette, IN 47907 USA; 2 Department of Comparative Pathobiology, College of Veterinary Medicine, Purdue University, West Lafayette, IN 47907 USA

**Keywords:** Canine, Fibrosis, Gastric muscularis layer, Ultrasonography, Unorganized hyperechoic striations

## Abstract

**Background:**

Ultrasonographic appearance of unorganized hyperechoic striations (UHS) has been observed in the canine gastric muscularis layer. The purpose of the study was to determine the prevalence, sonographic and postmortem histologic features, and to determine the clinical significance of canine gastric muscularis UHS. In the prospective study, 72 dogs were included. The presence of gastric muscularis UHS were reviewed to determine its distribution and location. In the retrospective study, 167 dogs that had both abdominal ultrasonography and necropsy were included.

**Results:**

The prevalence of gastric muscularis UHS in dogs was 37.5% in the prospective and 5.4% in the retrospective studies respectively. The higher prevalence in prospective study was due to greater anticipation by the radiologists in search for gastric muscularis UHS. In the ventral gastric wall, the muscularis UHS were better defined when the gastric lumen was empty or non-distended, and were mostly parallel with the serosa when the gastric wall was distended (with gas or fluid). Visualization of the dorsal gastric wall was often obscured by gas shadowing from luminal gas. Histopathology was performed on eight dogs with gastric muscularis UHS, three of which had fibrous tissue observed with Masson’s trichrome stain.

**Conclusion:**

Presence of gastric muscularis UHS in dogs may have been attributable to presence of incomplete interfaces between the inner oblique, middle circular and outer longitudinal layers of the gastric tunica muscularis or due to presence of fibrous tissue within the gastric muscularis layer. The clinical significance of canine gastric muscularis UHS is uncertain.

## Background

**Fig. 1:**
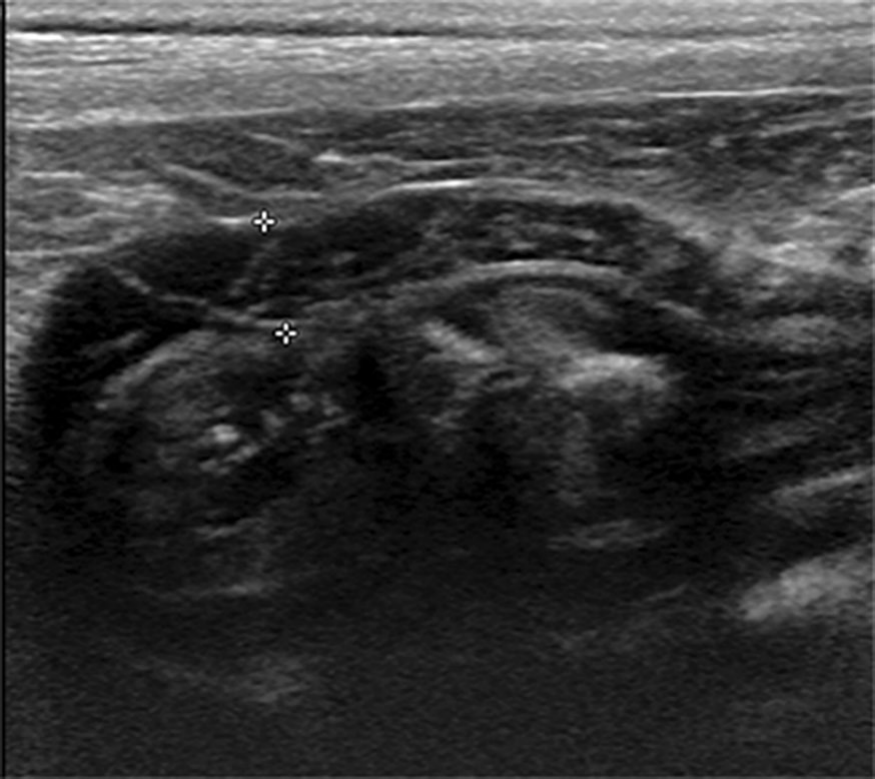
Sagittal ultrasonographic image of the stomach of a dog from the prospective study. The unorganized hyperechoic striations (UHS) are seen in the gastric muscularis layer (between the asterisks). This dog had no clinical sign related to the gastrointestinal tract

During routine ultrasonographic examination of the gastrointestinal tract (GIT), the GIT wall thickness and the 5-layered appearance, as well as the function (motility) are often assessed. The 5-layers of the stomach wall (from outermost to innermost) include the serosa, muscularis, submucosa, mucosa and mucosal surface. The alternating echogenicity appearance of these layers has been well-described in veterinary literature. The serosa, submucosa and mucosal surface are hyperechoic, while the muscularis and mucosa are hypoechoic [1]. The hyperechoic appearance of the mucosal surface is due to trapping of small gas bubbles at the mucosal surface and hence it is not considered as a true histologic layer [1]. Recently, hyperechoic bands paralleling the serosal layers of the muscularis layer of the canine colon wall has been reported and was found to be associated with the presence of fibrous tissue in the myenteric plexus or in the tunica muscularis [2]. Unorganized hyperechoic striations (UHS) have been observed within the canine gastric muscularis layer (Fig. 1) by the authors. This has never been reported and the clinical significance of this is unknown.

The aims of this study were to estimate the prevalence of canine gastric muscularis UHS, to characterize the ultrasonographic and histologic features, and to determine the clinical significance of canine gastric muscularis UHS wherever possible.

## Methods

This is a single institution, descriptive, cross-sectional study comprising of prospectively and retrospectively recruited sample populations of dogs.

In the prospective part of the study, all dogs that underwent routine abdominal ultrasound examination during a 4-week period (March 2014) were included. There were no exclusion criteria. The standard care of the institute was provided to each animal. All ultrasonographic studies were performed by two board-certified radiologists (HGH & CKL) using the same ultrasound system (Philips iU22 SonoCT system, Philips Ultrasound, Bothell, WA) and machine settings. Both linear (5–12 MHz) and micro-convex (5–8 MHz) transducers were used. The choice of the transducer used depended on the body conformation and size of the dogs. The selection of the location of the focus point varied depending on the type of transducer used. The linear transducer was preferred whenever possible for superior image resolution. This ultrasonographic examination was part of the standard care for the diagnostic evaluation of the patients, thus owner consent was not deemed necessary. The radiologists were not blinded and therefore aware of the clinical presentation of each dog that was presented for abdominal ultrasound.

The appearance of the gastric muscularis was evaluated to determine the presence of UHS. Still images and video clips were captured and reviewed by two board-certified radiologists (HGH & CKL) to determine the region of the stomach involved: (i) ventral wall, or (ii) dorsal wall, or (iii) both ventral and dorsal walls; and the distribution of UHS: (i) local if only one part of the fundus or body or pylorus in either of the ventral wall or dorsal wall is affected, or (ii) diffuse if more than one part of the fundus or body or pylorus of the ventral wall or dorsal wall is affected. Presence of gas or ingesta that may obscure visualization of the dorsal wall is also noted.

In the retrospective part of the study, all dogs that had both abdominal ultrasound and necropsy from January 2011 to December 2013 were included, without any exclusion criteria. All ultrasonographic studies were performed using the same ultrasound machine and settings used in the prospective part of this study and reviewed by both radiologists to identify dogs with gastric muscularis UHS. Due to the retrospective nature of the study and limited images of the stomach (usually two to three still images in both longitudinal and transverse planes) captured in routine abdominal ultrasound, the presence of gastric muscularis is remarked as present or not present. Once such cases were identified, the original histologic sections of formalin-fixed, paraffin-embedded stomach stained with hematoxylin and eosin (H&E) were reviewed by a board-certified veterinary pathologist (MAM). Additionally, Masson’s trichrome stain was used to identify fibrous collagen.

The medical records of dogs with gastric muscularis UHS in both the prospective and retrospective parts of the study including the signalment (gender, age and breed), clinical signs at presentation, previous or current history of GIT problems, current and pertinent laboratory results (including histologic results whenever available) and outcome were reviewed by a board-certified internist (SS) to determine if a dog had any underlying GIT disease.

## Results

**Fig. 2:**
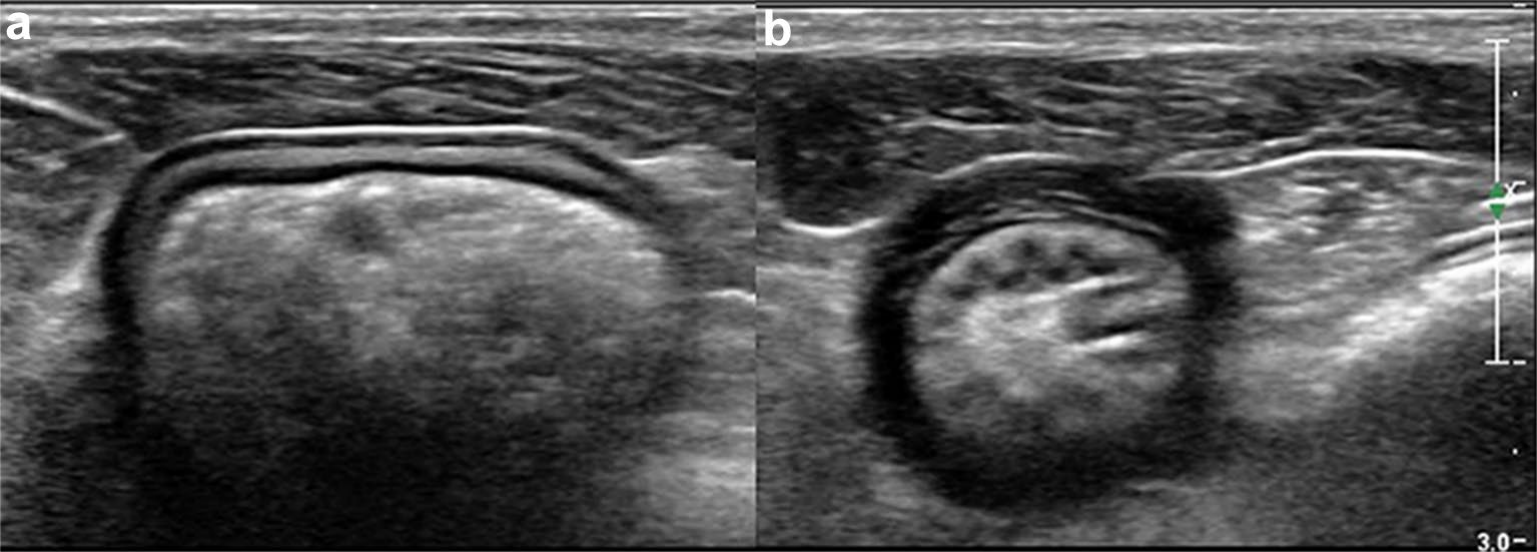
Transverse ultrasonographic images of one dog with the gastric muscularis UHS from the prospective study. The gastric muscularis UHS were subtle and appeared to be linear and parallel to the serosa when the stomach was relaxed (**a**) but were more prominent when the stomach was contracted (**b**)

A total of 72 dogs were evaluated during the prospective part of the study. Gastric muscularis UHS were observed in 27 dogs (37.5%). The entire stomach including the ventral wall and dorsal wall was visible in 10 dogs while only the ventral wall was visible for the remaining 17 dogs due to presence of intraluminal gas within the stomach. All 27 dogs with gastric muscularis UHS had diffuse distribution, involving all regions (fundus, body and pylorus) of the ventral and dorsal stomach wall that were visible. Gastric muscularis UHS were best defined when the gastric lumen was empty or non-distended (Fig. 2). This could be seen on both transverse and sagittal planes, using both micro-convex and linear transducers. The gastric muscularis UHS were subjectively better defined using linear transducer. The gastric muscularis UHS appeared to be parallel to the serosal layer when the gastric wall was distended with fluid or gas (Fig. 2A) but unorganized when the stomach is empty or non-distended. The 27 dogs with gastric muscularis UHS were comprised of 16 neutered female, 10 neutered male and one intact male with a mean age of 10 years 2 months (ranges from 3 years 2 months to 14 years 9 months). There were eight mixed breed dogs, two West Highland White Terriers, two Yorkshire Terriers and one of each of the following breeds: Papillion, English Springer Spaniel, Skye Terrier, Jack Russell Terrier, Greyhound, Rhodesian Ridgeback, Border Collie, Dachshund, Bernese Mountain Dog, English Bulldog, Golden Retriever, Cairn Terrier, Belgian Malinois, Pug and Bichon Frise. Six of 27 dogs with gastric muscularis UHS had some episodes of vomiting as part of their medical history. The vomiting could be attributed to the following underlying medical conditions (one each): acute or chronic renal disease, pyelonephritis, chemotherapy associated, splenic torsion, pyonephrosis, and hemoabdomen. None of the dogs had any evidence of chronic GIT disease. No other ultrasonographic abnormalities of the GIT were noted in these dogs. One other dog showed occasional soft stools likely attributable to receiving piroxicam for treatment of transitional cell carcinoma (TCC).

Still images from a total of 167 abdominal ultrasonographic examinations were reviewed for the retrospective part of the study. Based on the captured still images of the gastric wall available, presence of gastric muscularis UHS was observed only in nine out of the 167 dogs (5.4%), all of which were within the ventral wall only. The dorsal gastric wall was not visible due to intraluminal gas. The nine dogs were comprised of four neutered females and five neutered males with a mean age of 9 years 9 months (ranges from 5 to 15 years old) and were of the breeds Yorkshire Terrier (n = 2) and English Bulldog, Welsh Corgi, Chow Chow, Beagle, Australian Shepherd, Labrador Retriever (one of each breed) and a mixed breed dog. The nine individual dogs with gastric muscularis UHS and their respective significant findings are shown in Table 1. One dog (Dog 4) had neither clinical nor ultrasonographic signs related to GIT disease. Eight of nine dogs showed either clinical signs and/or ultrasonographic findings related to the GIT. In 4/8 dogs these findings were attributable to their primary disease process: gastric hematoma due to immune-mediated thrombocytopenia (Dog 1) progressive TCC and cholangio-hepatitis (Dog 3), liver failure (Dog 5), and glomerulonephritis and interstitial nephritis (Dog 8). In 2/8 dogs it was difficult to determine the reason for their GIT signs or ultrasonographic changes. Dog 6 was treated for multiple neoplastic diseases and also suffered from primary hyperparathyroidism and had an episode of acute kidney injury at the time of evaluation. This dog was found to have a ruptured hepatic adenoma leading to hemoabdomen. It was unclear what contributed most to his clinical signs, but there was no evidence for primary GIT disease. Dog 9 showed vomiting and lethargy of 12 h duration and multiple liver nodules on abdominal ultrasound. Necropsy was consistent with hepatic adenomas and pulmonary and renal amyloidosis was found concurrently. There was no evidence for primary GIT disease. In 2/8 dogs GIT disease could not be ruled out. Dog 2 suffered from septicemia and showed thickened gastric and duodenal wall on ultrasound. Necropsy confirmed acute pancreatitis with likely secondary enteritis. Dog 7 showed some weight loss on presentation and was diagnosed with a spinal meningioma and chronic kidney disease. In addition, mild eosinophilic enteritis was present.

**Table 1:** Dogs with gastric muscularis unorganized hyperechoic striations and their respective clinical and histopathological findings

Animal	Signalment	Clinical signs related to GIT disease	Primary disease process	Histopathology stomach
Dog 1	5 years FS Welsh Corgi	No	Immune-mediated thrombocytopenia	Light patchy (microscopic) mucosal and submucosal hemorrhage; no hemorrhage in muscularis layer
Dog 2	7 years FS Bulldog	Vomiting, hematemesis	Probable immune-mediated thrombocytopenia, DIC, septicemia	Diffuse light submucosal hemorrhage; patchy muscularis hemorrhage (light) and mainly along plexus and extending perivascular into inner and outer layers
Dog 3	15 years MN Yorkshire Terrier	Inappetance, melena	Transitional cell carcinoma, cholangio-hepatitis	Mucosal layer has accentuated deep follicles, but more diffuse lymphoplasmocytic superficial lamina proprial infiltration; parietal cells with swollen hypochromatic nucleus and cytoplasmic pseudoinclusion; cut perpendicular so looks different, but impression is increased mature fibrous tissue through tunica muscularis and serosa
Dog 4	8.5 years MN mixed breed dog	No	Glomerulopathy, cerebral infarcts	Normal mucosal layer; submucosal small arteries, arterioles, and veins have pale amphophilic homogeneous mural deposition (vasculopathy); surrounding fibrous tissue has pale basophilic coarse globular/stippling; inner layer muscularis has areas with drop-out of myofibers
Dog 5	8 years MN Chow Chow	Vomiting (medication related)	Hepatocellular necrosis with liver dysfunction, portal vein thrombosis	Normal mucosa/submucosa layers; increased fibrous tissue in muscularis; perivascular fatty infiltration in muscularis
Dog 6	15 years MN Beagle	Vomiting, diarrhea, anorexia	Acute kidney injury, primary hyperparathyroidism, and multiple neoplastic disease processes (transitional cell carcinoma, hepatocellular adenoma, mantle cell lymphoma, thyroid mass). Ruptured hepatic adenoma with hemoabdomen	No abnormal histopathologic findings
Dog 7	10.5 years FS Australian Shepherd	Weight loss	Spinal meningioma, chronic kidney disease and eosinophilic enteritis	No abnormal histopathologic findings
Dog 8	9 years MN Yorkshire Terrier	Weight loss	Renal tubular dysfunction, membranoproliferative glomerulonephritis with interstitial nephritis	Small portion of the outer layer of tunica muscularis has atrophied bundles); myenteric plexus and ganglia present; space between inner and outer layers expanded by edematous fibrous tissue with dilated lymphatics; submucosa and mucosa layer with mineralization of vessels and basement membrane; fibrin thrombi in mucosal venules (azotemia)
Dog 9	10 years FS Labrador Retriever	Vomiting	Hepatocellular adenoma, pulmonary and renal amyloidosis	No tissue

*FS* female spayed, *MN* male neutered

**Fig. 3:**
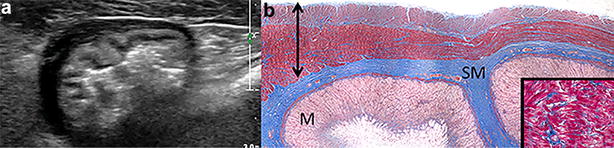
Transverse ultrasonographic image of a dog’s stomach (**a**) and corresponding histologic section with Masson’s trichrome stain (**b**) from the retrospective study. The gastric muscularis UHS were more prominent in the contracted portion of the stomach where the muscularis was thicker. In the histologic section, the fibrous tissue (blue) in the thicker part of the muscularis is mostly perivascular (see insertion at the right bottom corner), whereas in the thinner part, the blue-stained fibrous tissue is in the muscularis interstitium parallel to the muscle fibers and to the serosal surface (top). The double-headed arrow spans the muscularis. *M* gastric mucosa, *SM* gastric submucosa

Eight of these nine dogs had postmortem histologic examination of the stomach. Histologically, one dog (Dog 3) had mild lymphoplasmocytic gastritis and one dog (Dog 8) had mucosal mineralization. The muscularis layers of seven dogs were within normal limits in H&E-stained sections (including both dogs with mucosal changes), except for one dog that had increased mature fibrous tissue throughout the muscularis layer. With Masson’s trichrome stain, however, fibrous tissue was observed in the gastric muscularis layer in three dogs (Fig. 3). This change was mild to moderate and patchy in the two dogs without obvious fibrosis in H&E-stained sections, and more diffuse and extensive in the dog with apparent muscularis fibrosis in H&E-stained sections.

## Discussion

The higher prevalence of canine gastric muscularis UHS in the prospective study (37.5%) than in the retrospective study (5.4%) was expected because the prospective study was designed specifically to assess the presence of UHS in the gastric muscularis layer. In the prospective part of the study, multiple attempts were often made to capture optimal still images and video clips in order to demonstrate the presence of gastric muscularis UHS. In the retrospective part of the study, assessment for UHS in the gastric muscularis layer were only based on few (one or two) captured still images of the gastric wall, and some of these image quality may not have been optimized to illustrate the presence of gastric muscularis UHS. Gastric muscularis UHS were mostly observed in the ventral gastric wall because the evaluation of the dorsal gastric wall were often hampered by presence of artifacts originating from intraluminal gas. The gastric muscularis UHS and the diffuse distribution were best seen in a contracted (non-distended) gastric wall, possibly due to the increased thickness of the fibrous tissue (Fig. 2).

Based on the findings of this study, gastric muscularis UHS may be attributable to the presence of increased fibrous tissue within the muscularis layer. It is difficult to discern fibrous tissue in the gastric muscularis using conventional H&E-stain because smooth muscle and fibrous tissue have similar affinity for the eosin dye. Therefore, Masson’s trichrome stain was used in this study to differentiate blue-stained collagen fibers from red smooth muscle. With Masson’s trichrome stain, increased fibrous tissue was detected in three of the eight dogs where fibrous tissue was not detected with H&E stain. However, one may argue that fibrous tissue may not be the only possible explanation for presence of gastric muscularis UHS since five of the eight dogs did not have increased fibrous tissue within their tissue sections. The failure to detect an increase in fibrous tissue in the gastric muscularis in these five dogs could reflect sampling differences between the sonographic and the histologic examinations. Difficulties in a histologic search for sonographic lesions may also be due to small focal sample size (versus the entire stomach).

Perhaps a more plausible explanation for the appearance of the gastric muscularis UHS would be the unique nature of three sublayers within the normal canine gastric tunica muscularis: (i) inner oblique layer (ii) middle circular layer (iii) outer longitudinal layer [3, 4]. Presence of connective tissue or interface between these sublayers of the gastric tunica muscularis has been previously reported in humans and has also been corresponded to presence of additional thin hyperechoic layers within the gastric wall on ultrasound [5]. Similarly in veterinary medicine, presence of additional hyperechoic line within the muscularis layer of canine small intestines on ultrasound have also been corresponded to interface between longitudinal and circular layers of the tunica muscularis [6]. The hyperechoic striations appeared ‘unorganized’ because each of the sublayers of the gastric tunica muscularis were actually incompletely covering the stomach. For example, the outer longitudinal layer continues from the outer muscle of the esophagus, spreads widely over the pylorus but is thicker along the curvatures while the middle circular layer is distributed in hoops from the cardia to the pyloric canal [4]. The innermost oblique layer is very incomplete but compensates for the deficiencies in the circular layer as stout fascicles above the cardia and continuing distally to each side of the lesser curvature [4]. The advancement in ultrasonographic technology may have also contributed to increased feasibility of detecting gastric muscularis UHS. The image quality and resolution of the ultrasound equipment has improved tremendously compared to 20 years ago [7, 8]. Measurement of the individual layers of the GIT [9–11], detection of canine colonic muscularis hyperechoic band [2], small nodules in the submucosa layer of colon in dogs and cats [12] and mucosal fibrosis in cats [13] have been published recently due to the improvement of the resolution of ultrasound technology and equipment. In this study, the combination of improved resolution of the ultrasound machine with preferential use of high frequency linear transducer may have increased the likelihood of visualizing gastric muscularis UHS.

In the canine stomach, altered echogenicity of the muscularis layer has not been correlated with any specific disease. However, altered echogenicity of the mucosa layer has been associated with disease. A hyperechoic line at the gastric mucosal-luminal interface is usually secondary to mineralization in dogs with uremic gastropathy [14]. The presence of a gastric mucosal defect with accumulation of hyperechoic specks (microbubbles) is characteristic of gastric ulceration [15]. Presence of submucosal fat in feline stomach may lead to increased thickness and echogenicity of this layer [16, 17]. Fibrosis leading to presence of a linear hyperechoic band in feline mucosal layer has been reported [13]. In the prospective part of our study, 22% (7 of 27) of dogs showed clinical signs such as vomiting, inappetance or anorexia, diarrhea, or weight loss, which can be attributed to GIT disease. However, no dog was identified to have primary GIT disease and the clinical signs could be attributed to the primary disease process in each individual. Seven out of nine dogs in the retrospective part of our study were found to have clinical signs related to the GIT. However, all these animals were severely ill and most of them had multiple medical conditions potentially leading to GIT signs. Only three (Dogs 2, 3, and 7) out of eight dogs were found to have inflammatory changes of their stomach or intestines on histopathology. Dog 2 had mild duodenal and jejunal enteritis on histopathology. This dog was diagnosed with septicemia and pancreatitis, therefore the enteritis was considered more likely to be secondary to acute pancreatitis rather than primary GIT disease. In Dog 3, no clear association with primary GIT disease could be made. Dog 7 was found to have mild eosinophilic enteritis, which is most commonly seen either due to inflammatory bowel disease or parasitic infestation. The only sign possibly indicating GIT disease was weight loss and the dog was diagnosed with chronic kidney disease and a spinal meningioma. Primary GIT disease cannot be ruled out in this dog.

Canine gastric muscularis UHS were unlikely to be breed or gender specific as they were observed in large variety of canine breeds, ranging from small to large breed dogs, both in male and female. The mean age of the dogs with gastric muscularis UHS was about 10 years old. The lack of younger dog population in this study makes the correlation of age with canine gastric muscularis UHS impossible.

The small number of patients in which histopathology was available was a limitation of this study, though approximately 1/3 of the histopathologic samples evaluated had fibrous tissue present in the muscularis layer.

## Conclusions

This study is the first to describe the appearance of gastric muscularis UHS in dogs. Presence of gastric muscularis UHS in dogs may have been attributable to presence of incomplete interfaces between the inner oblique, middle circular and outer longitudinal layers of the gastric tunica muscularis or due to presence of fibrous tissue within gastric muscularis layer. Based on this study, this finding appears to have no significant clinical correlation with primary GIT disease.

## Abbreviations

GIT: gastrointestinal tract
H&E: hematoxylin and eosin
TCC: transitional cell carcinoma
UHS: unorganized hyperechoic striations

## References

[1] Penninck DG, Nyland TG, Fisher PE, Kerr LY (1989). Ultrasonography of the normal canine gastrointestinal tract. Vet Radiol.

[2] Heng HG, Lim CK, Miller MA, Broman MM (2015). Ultrasonographic observation of colonic muscularis hyperechoic band paralleling the serosal layer in dogs. Vet Radiol Ultrasound.

[3] Frappier BL, Eurell JA, Frappier BL (2006). Chapter 10 digestive system. dellman’s textbook of veterinary histology.

[4] Dyce KM, Sack WO, Wensing CJG (2002). The digestive apparatus. In textbook of veterinary anatomy, Chapter 3.

[5] Aibe T, Fuji T, Okita K, Takemoto T (1986). A fundamental study of normal layer structure of gastrointestinal wall visualized by endoscopic ultrasonography. Scand J Gastroenterol Suppl.

[6] Le Roux AB, Granger LA, Wakamatsu N, Kearney MT, Gaschen L (2016). Ex vivo correlation of ultrasonographic small intestinal wall layering with histology in dogs. Vet Radiol Ultrasound..

[7] Taxt T, Jirik R (2004). Superresolution of ultrasound images using the first and second harmonic signal. IEEE Trans Ultrason Ferroelectr Freq Control.

[8] Lin CH, Sun YN, Lin CJ (2010). A motion compounding technique for speckle reduction in ultrasound images. J Digit Imag.

[9] Donato PD, Penninck D, Pietra M, Cipone M, Diana A (2014). Ultrasonographic measurement of the relative thickness of intestinal wall layers in clinically healthy cats. J Fel Med Surg.

[10] Gladwin NE, Penninck DG, Webster CR (2014). Ultrasonographic evaluation of the thickness of the wall layers in the intestinal tracts of dogs. Am J Vet Res.

[11] Winter MD, Londono L, Berry CR, Hernandez JA (2014). Ultrasonographic evaluation of relative gastrointestinal layer thickness in cats without clinical evidence of gastrointestinal tract disease. J Fel Med Surg.

[12] Citi S, Chimenti T, Marchetti V, Millantra F, Mannucci T (2013). Micronodular ultrasound lesions in the colonic submucosa of 42 dogs and 14 cats. Vet Radiol Ultrasound.

[13] Penninck DG, Webster CR, Keating JH (2010). The sonographic appearance of intestinal mucosal fibrosis in cats. Vet Radiol Ultrasound.

[14] Grooters AM, Miyabayashi T, Biller DS, Merryman J (1994). Sonographic appearance of uremic gastropathy in four dogs. Vet Radiol Ultrasound.

[15] Penninck D, Matz M, Tidwell A (1997). Ultrasonography of gastric ulceration in the dog. Vet Radiol Ultrasound.

[16] Heng HG, Teoh WT, Sheikh Omar AR (2008). Gastric submucosal fat in cats. Anat Histol Embryol.

[17] Heng HG, Wrigley RH, Kraft SL, Powers BE (2005). Fat is responsible for an intramural radiolucent band in the feline stomach wall. Vet Radiol Ultrasound.

